# Molecular docking analysis of human JAK2 with compounds from tomatoes

**DOI:** 10.6026/97320630016742

**Published:** 2020-10-31

**Authors:** Umapathy Vidhya Rekha, M Anita, S Bhuminathan, K Sadhana

**Affiliations:** 1 Department of Public Health Dentistry, Sree Balaji Dental College and Hospital, Pallikaranai, Chennai 600 100, India; 2 Department of Prosthodontics, Sree Balaji Dental College and Hospital, BIHER, Pallikaranai, Chennai 600 100, India

**Keywords:** Lycopersicon esculentum, JAK2, Oral Cancer, Molecular docking

## Abstract

Janus kinase 2 (JAK2) is a tyrosine kinase receptor that belongs to the JAK family kinases is linked to oral cancer. We describe the molecular binding analysis of JAK2 with 23 compounds from tomotoes. Docking data shows five compounds (rutin, qucertin, narigenin, chlrogenia acid & kaempferol) with optimal binding features with JAK2 for further consideration.

## Background

Oral cancer is the 6th frequently occurring cancer between both male and female population, and the third most common cancer in developing nations [1]. The majority of oral cancers are known as squamous cell carcinoma [2,3], which are malignant and responsible to develop rapidly. In India, oral cancer ranked as first place among all other types of cancer in males and third commonest cancer between females in various regions [4]. Common reason for this oral cancer is tobacco and alcohol. Evading of tobacco and alcohol is the most significant precautionary action against mouth, throat and lung cancers. Oral cancer can be identified in early stage through the close interaction of the peoples who have habit of tobacco [5]. The discovery of toxic free, effective treatment, with complementary and alternative therapies, is serious if the survival rate is to be increased. Epidemiologic studies have proposed a defensive result from some plant-derived foods and extracts [6]. Many epidemiological reports proposed that the eating of tomatoes (Lycopersicon esculentum) decreases the risk of cancer [7]. There are many recent reports suggested that regular consumption of small amount of tomato products used to protect the cell from DNA damage in oxidant species [8]. Because of notorious values, the tomatos have their antioxidant and antitumoral properties. Computer aided drug design is one of the fastest drug designing methods; it includes various methods to discover novel compounds. One of such method is molecular docking study of drug with target protein [9]. Molecular docking is one of the best methods used to identify the orientation of compounds to the target receptor to facilitate the binding affinity and activity of the small molecules.

The Janus kinase (JAK) belongs to the family of family of tyrosine kinases and contains four members such as tyrosine kinase 2, JAK1, JAK2, JAK3, and functions as a regulator of signaling pathways activated by a number of growth factor and cytokines [10]. Among them, JAK2 kinase plays key roles many neoplastic diseases and is extremely expressed in numerous cell types [10]. Activation of the JAK2/Signal transducer and activators of transcription 3 (STAT3) signaling pathway has revealed to have vital roles of tumorigenesis and progression in different human tumor cell types [11,12]. Therefore, the blockade JAK2/STAT3 signaling pathway inhibits cell proliferation and provokes apoptosis of numerous human cancer cells [13]. More exclusively, it has been reported that cell growth is suppressed by interference with JAK2/STAT3 signaling in OSCC [14]. So, in the present study we collected the available compounds from tomato (Table 1 - see PDF) and identified their effect against oral cancer target JAK2 using molecular docking approach.

## Materials & Methods:

### Protein Preparation:

The 3D crystal structure of Janus Kinase 2 was downloaded from PDB with PDB code (2B7A) is downloaded from PDB and processed adequately for further analysis [15-17].

### Ligand Preparation:

We used 12 reported compounds from tomato plant from literature. The structures of these compounds were retrieved in the Spatial Data File (.SDF) file format from the PubChem Compound Database (National Center for Biotechnology Information at https://pubchem.ncbi.nlm.nih.gov/). All the structures were converted from .SDF to PDB format with the help of the online smiles translator. PDB format were then converted to the ligand PDBQT format using ADT for use in AutoDock4 (AD4) and Auto Dock Vina [18]. AutoDock Vina was for the docking studies of compounds with the target JAK2 receptor [18]. Docked receptor-ligand complexes were visualized using PyMOL. It showed the active site, hydrogen-bond interactions, hydrophobic interactions, and bonding distances as interaction radii of the docked ligand. The binding poses of all compounds were observed and their interactions with the JAK2 were characterized, and the top most energetically good conformations of every ligand were selected.

## Results and Discussion:

A molecular docking study was carried out to identify the biological activity of compounds from tomato against the JAK2 receptor in oral cancer. For the selected compounds and protein the docked binding mode was recognized to link the docking score function. The binding pattern analysis among JAK2. Protein and ligands recommended that the binding pattern diverse with the ligand nature.

Protein –ligand interaction happen naturally only if the free energy change is negative and the variation in ∆G levels of complexed and unbound free states is proportional to the stability of the protein–ligand interaction. It follows that both protein folding and protein–ligand binding occur when ∆G is low in the system [19,20]. So negative ∆G scores showed the stability of docked protein-ligand complexes, and it is important feature for effective drugs [21]. In the present study, rutin– JAK2 complex had the more negative ΔG values, so this indicates that rutin have high binding affinity towards the target protein JAK2. Results of all other compounds also had good binding affinity with selected receptor in terms of low binding score. Molecular docking studies also used to identify the types of binding like hydrogen bond, hydrophobic, and electrostatic interactions, with essential amino acid residues are indicative of ligand docking in favorable conformations [22]. Among them hydrogen bond are the main contributors to the stability of receptor protein.

Hence, in the present study, results of docking showed that hydrogen bond, hydrophobic and electrostatic interactions are mediated through different amino acid residues in each ligand–protein interaction. Specially, the amino acids GLU -930, LEU 932 & LYS-857 alternatively form the H bond with most of the compounds. Out of 12 compounds were selected and showed in Table 2 (see PDF).

**Figure 1:**
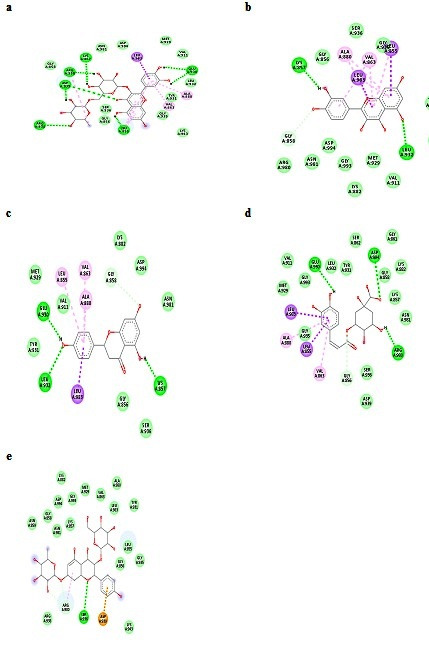
Molecular docking analysis of JAK2 with (a) rutin, (b) qucertin, (3) narigenin, (4) chlrogenia acid and (5) kaempferol

Compared to other compounds rutin formed six H bond interaction (LEU-855, ARG- 938, ASP-939, ARG-980, LYS-857 & GLU-930) with JAK2 receptor this was showed in Table 2 (see PDF) and can be seen in Figure 1a. Presence of Pi-sigma (LEU-983) and Pi-alkyl (ALA-880; VAL-863) interactions mainly participated in charge transfer of molecules and also helped to intercalating the drug in the active site of the Target protein (Figure 1a). The compound Qucertin interact with JAK2 receptor molecule satisfactorily with good docking score of -9.3 kcal/mol, making it the second most active drug. It's showed two H-bonds with LYS-857 & LEU-932 respectively (Figure 1b). Further Qucertin also form Pi-sigma interaction with LEU -855 & LEU-932 and pi-alkyl interaction with ALA-880 & VAL-863amino acids residues. Narigenin docked well with the JAK2 receptor with binding score of -8.9 kcal/mol. Three H-bonds were recognized between the JAK2 and Narigenin molecule. Narigenin formed the H bond with LYS-857, GLU-930 & LEU-932 amino acids residues of JAK2 protein. In addition, Leu-983 formed the Pi-sigma bond and LEU-855 & ALA-880 form pi-alkyl interaction with the receptor JAK2 (Figure 1c).

Chloregenic acid also showed efficient binding with the JAK2 receptor having a docking score of -8.2 Kcal/mol. It formed the three H-bond interactions with amino acids GLU -930, ASP- 994 & ARG -980 respectively. The docked complex stability also connected with extra Pi-sigma interaction (LEU-855 & LEU-983) and Pi-alkyl interactions (ALA-880 & VAL-863). All these interaction were shown in Figure 1D. Results of docking studies showed that binding score of Kaempferol with the JAK2 receptor is -7.4 Kcal/mol, this docked complex was achieved by one H bond interaction with SER-936 amino acid and one Pi-Sulfur interaction with ASP-939 and one Pi-alkyl interaction with ASP-939 (Figure 1E). All these interactions are induced the stabilizing charges responsible for intercalating the compound within JAK2 receptor. These types of interactions are also responsible for the shape of the docked complex.

## Conclusion

We describe five compounds (rutin, qucertin, narigenin, chlrogenia acid & kaempferol) with optimal binding features with JAK2 for further consideration.

## References

[1] La Vecchia C (1997). Oral Oncol.

[2] Elango JK (2006). Asian Pac J Cancer Prev.

[3] Coleman MP (1993). IARC Sci Publ.

[4] Parkin DM (1997). IARC Sci Publ.

[5] Zhang Y (2019). Tob Induc Dis.

[6] Pandey KB (2009). Oxid Med Cell Longev.

[7] Sesso HD (2003). J Nutr.

[8] Riso P (2004). Eur J Clin Nutr.

[9] Meng XY (2011). Curr Comput Aided Drug Des.

[10] Verma A (2003). Cancer Metastasis Rev.

[11] Sen M (2015). Neoplasia.

[12] Xiong H (2008). Neoplasia.

[13] Jia L (2016). PloS one.

[14] Peng H-Y (2016). PloS one.

[15] Morris GM (2009). J Comput Chem.

[16] Lim SV (2011). BMC Bioinformatics.

[17] Jaghoori MM (2016). J Comput Aided Mol.

[18] Trott O, Olson AJ (2010). J Comput Chem.

[19] Sergeev YV (2014). Curr Protoc Protein Sci.

[20] Du X (2016). Int J Mol Sci.

[22] Hariono M (2016). Sci Rep.

